# Investigation of a New Handover Approach in LTE and WiMAX

**DOI:** 10.1155/2014/246206

**Published:** 2014-10-14

**Authors:** Mohammad Nour Hindia, Ahmed Wasif Reza, Kamarul Ariffin Noordin

**Affiliations:** Department of Electrical Engineering, Faculty of Engineering, University of Malaya, 50603 Kuala Lumpur, Malaysia

## Abstract

Nowadays, one of the most important challenges in heterogeneous networks is the connection consistency between the mobile station and the base stations. Furthermore, along the roaming process between the mobile station and the base station, the system performance degrades significantly due to the interferences from neighboring base stations, handovers to inaccurate base station and inappropriate technology selection. In this paper, several algorithms are proposed to improve mobile station performance and seamless mobility across the long-term evolution (LTE) and Worldwide Interoperability for Microwave Access (WiMAX) technologies, along with a minimum number of redundant handovers. Firstly, the enhanced global positioning system (GPS) and the novel received signal strength (RSS) prediction approaches are suggested to predict the target base station accurately. Then, the multiple criteria with two thresholds algorithm is proposed to prioritize the selection between LTE and WiMAX as the target technology. In addition, this study also covers the intercell and cochannel interference reduction by adjusting the frequency reuse ratio 3 (FRR3) to work with LTE and WiMAX. The obtained results demonstrate high next base station prediction efficiency and high accuracy for both horizontal and vertical handovers. Moreover, the received signal strength is kept at levels higher than the threshold, while maintaining low connection cost and delay within acceptable levels. In order to highlight the combination of the proposed algorithms' performance, it is compared with the existing RSS and multiple criteria handover decision algorithms.

## 1. Introduction

With rapid development and deployment of wireless technologies (WiMAX, LTE), mobile networks should provide full mobility for all mobile stations simultaneously and, at the same time, guarantee the required quality of services. One of the main challenges of seamless mobility is the availability of efficient horizontal and vertical handovers (HHO, VHO). The handover which occurs between two networks using the same technology is called horizontal handover (HHO), for instance, WiMAX-to-WiMAX or LTE-to-LTE handovers [1], whereas the handover occuring between different technologies is called vertical handover (VHO), for instance LTE-to-WiMAX handover or vice versa [2].

Handover process experiences many obstacles, such as to predict an accurate target base station (BS) and to select an appropriate technology to connect with. Furthermore, complex calculations are required during the selection process of the target BS and technology from the BSs suggestion list [3]. The selection process between technologies should be accurate and able to satisfy the user's preferences. Otherwise, the mobile station (MS) keeps Ping-Ponging between technologies to search for better connection. The Ping-Pong effect causes unnecessary handoff processes and brings some weaknesses, including low network throughput, long handoff delay, and high dropping probability [4, 5]. Another issue is the two interferences: the intercell interference (ICI) and the cochannel interference (CCI) from the surrounding BSs, which sharply degrades the received signal strength [6–8]. In some cases, the interferences surpass the acceptable levels leading to connection loss between the MS and BS. The interference avoidance scheme is proposed to reduce the interferences from neighboring BSs by avoiding collisions between similar frequencies used by neighboring BSs. This goal can be achieved either in a static manner, which allocates different frequencies to neighboring BSs (such as frequency reuse factor), or in an intelligent way which adjusts the cells' radius based on the interference level. Considering the signaling overhead and complexity in implementing the intelligent technique, only the static method is widely adopted. These issues inherit the connection between the MS and BS, such as increasing the data loss rate, end-to-end delay, and connection cost.

Recently, several handover prediction methodologies have been proposed to predict and select the target BS with proper technology network, namely,

1. a history prediction approach that depends on the user's history and current location: firstly, the user mobility's history and handover ratio from the serving to the target BS are recorded and secondly, the prediction is performed based on the frequency of previous handovers between the serving and the target BS [9]. Some of the drawbacks of such methods are the fact that they are more suitable for the static nature of MS' mobility, and the target BS has to be stored in the user's mobility history;
2. another prediction approach that is based on determining the exact MS locations to predict the target BS so that MS could handover itself, that is, GPS [10, 11]: this approach provides an accurate prediction of the target BS but is still insufficient for certain cases in terms of high cost, long time process, and high power consumption;
3. an important improvement in the handover prediction that utilizes the received signal strength for both target and serving BSs is proposed in [12]; this approach does not require any parameters, except those for the RSS' measurements from the surrounding BSs. Once the RSS of the serving base station decreases below a predefined threshold level; the MS shifts to the neighboring BS. This approach suffers from signal attenuation, such as fading and shadowing, that is, where the handover prediction probability decreases as the signal attenuation increases.

Many algorithms have been proposed for vertical handover procedure based on a variety of criteria, such as available bandwidth, received signal strength, signal to inference ratio (SIR), connection cost, handover delay, MS' velocity, battery consumption, and quality of service (QoS). For instance, in [13], the MS checks the surrounding BSs and then hands over to the one which can offer the lowest delay and the highest bandwidth. Therefore, it is clear that it is not possible to make an appropriate handover decision only by evaluating those criteria (delay and bandwidth) leading to an increase in the wrong handover prediction ratio, whereas in [14], the handover technique offers a less complex algorithm while maintaining a robust VHO decision among heterogeneous networks. Furthermore, this method is based on evaluating the multiple criteria received from the neighboring BSs and determines the potential target BS. These criteria are the available bandwidth, cost of service, received signal strength, expected time to stay in practical network, and power consumption. Despite the fact that this approach shows high efficiency in low interference environments, it cannot work properly in high interference areas.

From the attenuation side, several studies have successfully decreased the interference from the surrounding BSs and efficiently utilized the available frequency spectrum by avoiding the collisions between the similar operating frequencies. This avoidance is achieved by several approaches and techniques. For example, in [15], the authors propose a new method to determine a proper frequency operation for each one of the inner cell, outer cell, and femtocell, whereas in [16], the ICI cancellation is realized using the biorthogonal frequency division multiple access cellular system along with multiple angle division reuse scheme. Moreover, another technique is reported in [17]; it suggests a novel dynamic interference cancellation method based on two levels. In the first level, it determines the coordination of each intercell in the network; then, at the second level, the central controller allocates the most appropriate chunk for the user terminal, which causes no conflict between the terminals. Furthermore, in [18], a method based on the power control and proper reuse of the frequency offers an attractive solution to the margining problem between the high power node (main base station) and low power node (relay node) in the same network.

Up till now, the most efficient method to eliminate the interferences in LTE technology is proposed in [6]. The proposed mechanism divides the cell into two regions, the inner and outer region, and selects the optimal size as well as the optimal frequency allocation between these regions. However, this technique seems to be insufficient to cooperate with other handover approaches due to the setup time and operation procedures that add much delay compared to static ones (fractional frequency reuse ratio technique). The FRR3 (frequency reuse ratio 3) exhibits an acceptable level of efficiency in terms of interference reduction from surrounding BSs while maintaining system simplicity better than the one reported in [6]. The authors have demonstrated a target BS prediction mechanism and technology selection method based on the user's preference without taking into consideration the prediction scenarios and the investigation of the FRR3 technique [19].

In this paper, we propose and demonstrate an enhancement on the existing target BS prediction algorithms (GPS and RSS) by introducing a virtual trigger threshold. The implementations of the two enhanced algorithms allow the user to tradeoff between predictions' criteria, namely, cost, power consumption, and accuracy. Furthermore, a modified multiple criteria with two thresholds algorithm is suggested to permit the user to select the target technology (WiMAX or LTE) based on its priorities, such as connection cost, delay, available bandwidth, and received signal strength. Moreover, the combination of target BS prediction approach, technology selection approach, and FRR3 technique is reported in this paper for the first time.

The rest of this paper is organized as follows.  Section 2 describes the system model, which consists of the enhanced GPS and novel RSS prediction approaches. Moreover, the selection procedure of appropriate technology and the overview of FRR3 with related equation are also discussed and elaborated in detail.  Section 3 contains a detailed study of the performance evaluation of the proposed approaches. Finally, the conclusion and the related future work are presented in Section 4.

## 2. System Model

**Figure 1:**
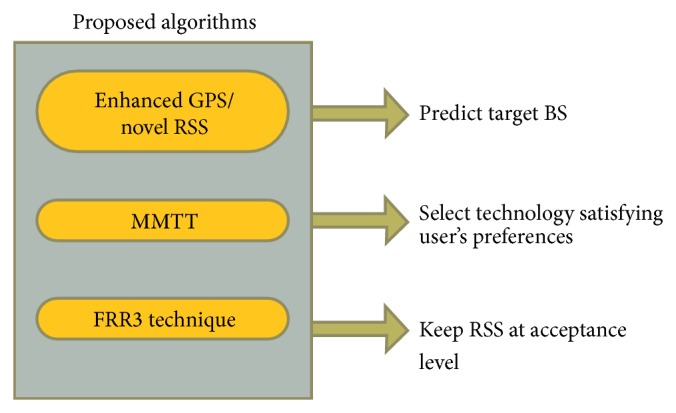
The system model.

As shown in Figure 1, the proposed algorithms are divided into three stages. In the first stage, based on the user's preferences, either enhanced GPS or novel RSS is selected to predict the target BS. In the next stage, modified multiple criteria with two handover thresholds (MMTT) algorithm selects the most appropriate technology (LTE or WiMAX) which satisfies the user's preferences. Finally, FRR3 technique decreases the ICI and CCI interferences from the surrounding BSs.

### 2.1. Prediction Approaches of the Target BS

**Figure 2:**
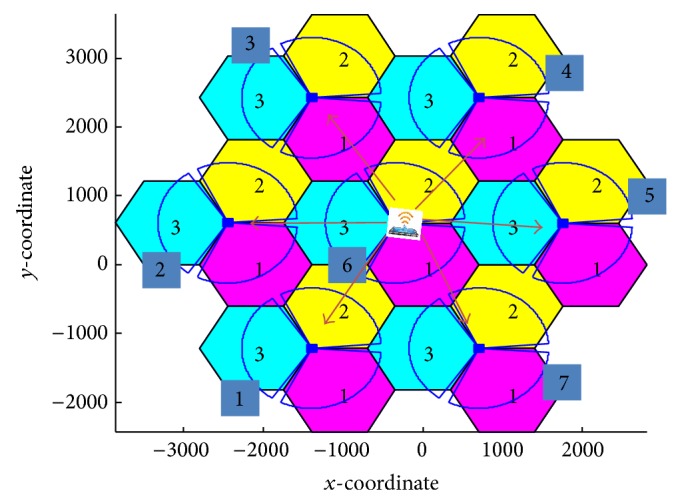
Potential movements of the MS.

Figure 2 illustrates the necessity behind applying the prediction approaches. Due to the random movement of the MS, its suggestion list combines 6 possible BSs as a target BS. Each BS offers two technologies (LTE and WiMAX); thus, the MS should go through 12 options as a searching process for the optimal connection as follows: {(1, LTE), (1, WiMAX),…, (6, LTE), (6, WiMAX)}. The introduction of prediction approaches significantly reduces the suggestion list to a maximum of two BSs with four possibilities. Therefore, at least 60% of the search process is reduced out of the calculations. Consequently, a sharp decrease in probability of connection loss, prediction time, and system complexity is observed. In the following subsections, two efficient prediction approaches are proposed, namely, enhanced GPS and novel RSS prediction approaches.

#### 2.1.1. GPS Prediction Approach

The enhanced GPS prediction approach is subject to MS' behavior, such as angle of movement, cross-distance, and velocity. Once the MS' RSS reaches the trigger threshold level (trigger threshold is the proposed virtual RSS level located at level +10 dBm higher than handover threshold), the GPS device is activated to determine the current coordination in the layout of three dimensions (latitude (*x*), longitude (*y*), and ellipsoid height (*z*)). Then, the coordination is kept updating up to *n*-time intervals (*n* is the range between the trigger and handover threshold). For each coordination measurement, the crossed distance is calculated as the difference between the current and previous MS' location during one time interval (1). Also, the angle of movement is determined based on the *x*-axis as 0-degree:

(1) D(n)=((x(n)−x(n−1))2+(y(n)−y(n−1))2  +(z(n)−z(n−1))2)1/2,

where *D*(*n*) is the MS crossed distance, (*x*(*n* − 1), *y*(*n* − 1), *z*(*n* − 1)) is the MS' coordination at *n* − 1 time interval, and (*x*(*n*), *y*(*n*), *z*(*n*)) is the MS' coordination at *n*-time interval.

The MS' velocity (*v*) is the MS crossed distance divided by the required time to cross it (*t*) (2):

(2) $$\begin{array}{c} v=\frac{D\left(n\right)}{t}. \end{array}$$

By calculating the movement angle, crossed distance, and velocity, the next BS is predicted accurately. The main enhancements added to GPS approach are power consumption and prediction cost along with high prediction accuracy, since the GPS device is activated during *n*-time intervals (between the trigger and handover thresholds) instead of keeping the GPS device on all the time.

#### 2.1.2. RSS Prediction Approach

The main objective of the novel RSS approach is to foretell the target BS in lower cost, in a simpler and faster way than the existing algorithms. It does not require any additional information, neither from the BS nor from the MS. MS' RSS is measured frequently for *n*-time intervals (from trigger to handover thresholds) from all surrounding BSs (3). Then, the highest RSS accumulative value is set as the target's BS (4). The robustness of the approach is that, it takes *n* RSS measurements instead of one RSS measurement, so, even if the interferences blur the prediction decision for a while, the target BS keeps maintaining the highest RSS accumulative value which is higher than others. The RSS is calculated as the following:

(3) $$\begin{array}{c} {\text{RSS}}_{i}\left(\text{MS}\right)={\text{PT}}_{i}+\text{GR}+{\text{GT}}_{i}-{\text{PL}}_{i}-{\text{LT}}_{i}-\text{LR}, \end{array}$$

(4) $$\begin{array}{c} \text{tar}{\text{BS}}_{i}=\text{MAX}\overset{n}{\underset{j=1}{\sum }}\overset{N}{\underset{i=1}{\sum }}{\text{RSS}}_{i,j}\left(\text{MS}\right), \end{array}$$

where RSS_*i*_(MS) represents the RSS received at the MS from BS_*i*_, the transmission powers of BS_*i*_ (PT_*i*_), GR, and GT_*i*_ are the antenna gain of both MS and BS_*i*_, respectively, PL_*i*_ is the path loss between BS_*i*_ and MS, LR and LT_*i*_ are the thermal receivers noise in both MS and BS_*i*_, respectively, tarBS_*i*_ represents the target base station *i*, *n* is the time interval between the trigger and handover threshold, *j* is the interval time index, and *N* is the total number of BSs.

### 2.2. MMTT Approach to Select the Accurate Target Technology

The MMTT approach is proposed to determine the most appropriate target technology (WiMAX or LTE), which can satisfy the user's preferences. This approach is based on evaluating many criteria, such as RSS, connection cost, handover process delay, and offered bandwidth. The MMTT guarantees stability of the connection by constantly maintaining the MS' RSS at an acceptable level and minimizing the number of redundant handovers between technologies due to the network selection, which depends on the user's preferences. The MMTT approach is illustrated as follows.

#### 2.2.1. Handover RSS Threshold and Triggered RSS Threshold Calculations

While the MS is moving across cells, it keeps tracking the RSS' serving BS. Once it equals the RSS trigger threshold level, the selection process of the most appropriate target technology starts. If the MS' RSS of technology *k* at serving BS_*x*_ is less than the handover threshold and the MS' RSS of technology *k* at target BS_*y*_ is bigger than or equal to the handover threshold, then the RSS condition is satisfied:

(5) |RSSm,k,x<RSSth,x|,|RSSm,k,y≥RSSth,y|,

where RSS_*m*,*k*,*x*_ is RSS received at the MS_*m*_ from the technology *k* at service BS_*x*_, and RSS_*m*,*k*,*y*_ is RSS received at the MS_*m*_ from the technology *k* at target BS_*y*_, while RSS_th,*x*_ and RSS_th,*y*_ are the handover thresholds for the service BS_*x*_ and target BS_*y*_, respectively. To increase the accuracy of handover and trigger thresholds for both serving and target BSs, a self-learning algorithm is developed, as shown below:

(6) $$\begin{array}{c} {\text{RSS}}_{\text{th},x}=\frac{1}{r}\times \overset{l}{\underset{i=1}{\sum }}{\text{RSS}}_{m,x,k}^{i},\\ {\text{RSS}}_{\text{th},y}=\frac{1}{r}\times \overset{l}{\underset{i=1}{\sum }}{\text{RSS}}_{m,y,k}^{i}, \end{array}$$

where *r* is the number of previous handover processes that occur between the serving BS_*x*_ and target BS_*y*_, *i* is the index of the handover event, and RSS_*m*,*x*,*k*_^*i*^ and RSS_*m*,*y*,*k*_^*i*^ are the RSS measurements at the MS_*m*_ from the serving BS_*x*_ and target BS_*y*_, respectively, at handover event.

The self-learning algorithm is triggered after two handover events, and then it keeps calculating and updating the threshold values of serving and target BSs up to *n*-time intervals. The main enhancement added by self-learning algorithm is the determination of the most accurate handover and trigger threshold values experimentally, which helps to prevent the sudden disconnections, especially in a high attenuation area, since the threshold values are set to be dynamically adopted with the surrounding area.

#### 2.2.2. The Technology Selection Process

The MS movement direction of the BS' technologies (WiMAX and LTE) will be evaluated by Algorithm 1; one of the technologies is selected as the accurate target technology for handover. Each technology has to satisfy four conditions to be added to the target technology suggestion list as follows.

1. The RSS of evaluating technology (RSS_*k*_) is bigger than the RSS threshold (RSS_th_) (Algorithm 1 (line 7)).
2. The technology connection cost (*C*_*k*_) is less than the connection cost threshold (*C*_th_) (line 8).
3. The technology handover process delay (*d*_*k*_) is less than the delay threshold (*d*_th_) (line 9).
4. The technology offered bandwidth (*b*_*k*_) is bigger than the bandwidth threshold (*b*_th_) (line 10).

Finally, the user will commence handover to the best technology *k* in BS_*i*_, which can provide the maximum value of (7) (line 15):

(7) TQE=WC×(1/Cmax⁡⁡(1/C))+WB×(Bmax⁡⁡B)+WD×(1/Dmax⁡⁡(1/D))+WRSS×(RSSmax⁡⁡RSS),

where TQE is the technology quality evaluation, WC, WB, WD, and WRSS are the weights of connection cost, available bandwidth, delay, and received signal strength, respectively, max (1/*C*), max *B*, max (1/*D*), and max RSS are the maximum values of connection cost, available bandwidth, delay, and received signal strength, respectively, among the technologies in the suggestion list.

The technologies added to the suggestion list should be validated by these steps. Then, the highest TQE value will be set as the target technology. It is worth mentioning that the sum of the weights is 100%.

### 2.3. FRR3 Technique

The main objective behind utilizing the FRR3 is decreasing the CCI and ICI interferences in order to significantly improve the received signal strength and the handovers' stability (VHO and HHO). The FRR3 maintains mutual interference between MS and surrounding BSs below a harmful level, especially at a high attenuation area. This guarantees a good performance of the MS-BS connection and the handover process. Moreover, FRR3 is simple and easy to apply. The FRR3 technique divides WiMAX and LTE BSs into 3 sectors and 3 hexagons, respectively. Each BS' sector and hexagon has its unique operating frequency, taking into consideration the minimum frequency reuse distance (dm). As a result, the ICI and CCI interferences sharply decrease compared to those without applying the FRR3 technique since the interferences come only from the adjacent sectors and hexagons in the same propagation angle (using the same frequency operation) instead of from all surrounding BSs, as illustrated below [6]:

(8) $$\begin{array}{c} {\text{SINR}}_{m,f}=\frac{{\text{PL}}_{i,m}\times T_{i,f}\times h_{i,m,f}}{\sigma_{f}^{2}+\sum_{S=1}^{y}I_{S,x}\times T_{S,f}\times h_{S,x,f}}, \end{array}$$

where SINR_*m*,*f*_ is the signal-to-interference-plus-noise ratio, PL_*i*,*m*_ refers to the path loss associated with the channel between MS_*m*_ and BS_*i*_, *T*_*i*,*f*_ is the transmit power of the BS_*i*_ on subcarrier *f*, *h*_*i*,*m*,*f*_ is the exponentially distributed channel fast-fading power, *σ*_*f*_^2^ is the noise power of the Additive White Gaussian Noise channel, *S* is the BS index, and *y* is the number of cochannel BSs.

## 3. Results and Discussion

To clarify the enhancement of the previous proposed algorithms, prediction and the overall scenario are tested by the NS-2 simulator and MATLAB program. The prediction scenario is validated at the University of Malaya area. The coordination data have been collected from the Google Map database and called to simulate inputs. The predicting target BS using the enhanced GPS and novel RSS prediction approaches is presented in Sections 3.1.1 and 3.1.2, respectively, and compared with the existing RSS approach, whereas the overall scenario is presented in Section 3.2 and evaluated in terms of target BS prediction, HHO and VHO, RSS, delay, and cost. Then, it is compared with the existing RSS and multiple criteria approach.

### 3.1. Prediction Scenario

**Figure 3:**
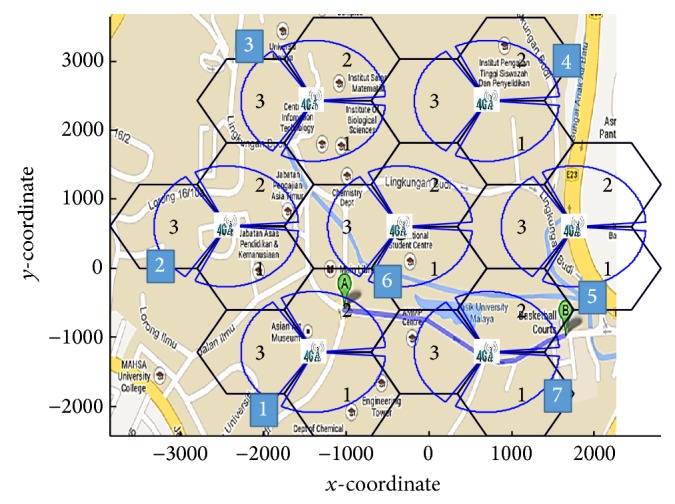
MS movement at the University of Malaya area.

The two prediction approaches are applied and tested at the University of Malaya area, as shown in Figure 3. The MS is assumed to be roaming from point A, located in BS 1, to point B, located in BS 7. These BSs are supported with LTE and WiMAX technologies.

#### 3.1.1. Enhanced GPS Approach Results

**Figure 4:**
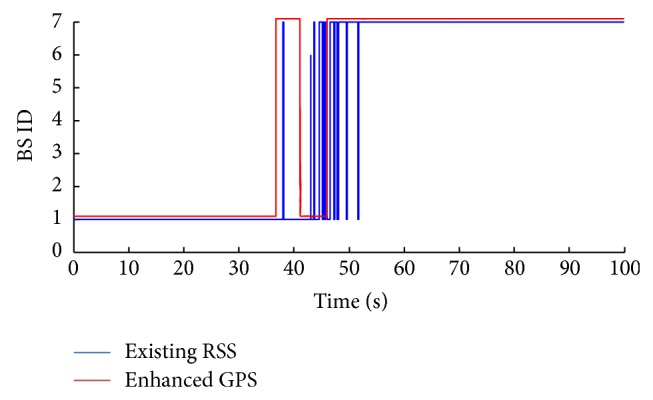
BS prediction using enhanced GPS prediction approach.

Once the MS reaches the trigger threshold level of the BS 1, the GPS device is activated. The GPS prediction approach determines MS' coordination (*x*, *y*, and *z*) and keeps tracking the coordinates up to the handover threshold (during *n*-time intervals), as illustrated in Section 2.1.1. In Figure 4, the enhanced GPS prediction approach proves a high prediction quality compared to the existing RSS approach [12]. Between 38 and 52 seconds, the existing RSS approach shows inaccurate and unstable determination of the target BS, since high attenuation of BS' edges lead to fluctuation in RSS level, which makes the BS prediction decision unclear, and the MS is continuously shifted between BS 1 and BS 7, which leads to a high ratio of Ping-Pong effects. In terms of the handover accuracy ratio (the proportion of the number of accurate handovers over the total number of handovers), the enhanced GPS shows a high level of accuracy compared to the existing RSS, with only two missed handovers for the enhanced GPS.

#### 3.1.2. Novel RSS Prediction Approach Results

**Figure 5:**
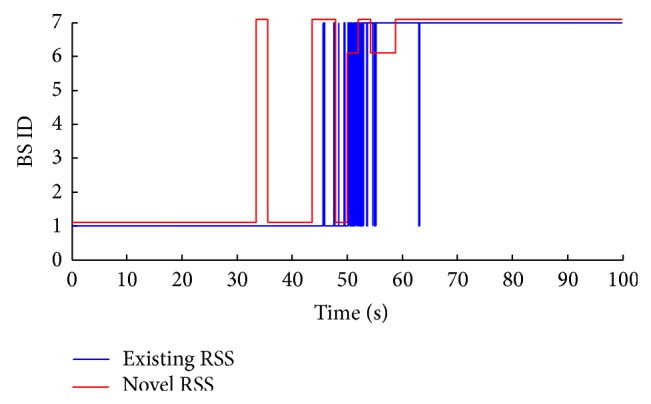
BS prediction using RSS prediction approach.

In order to demonstrate the improvement of the novel RSS prediction approach, it is compared to the existing RSS prediction approach in [12] (Figure 5). From 45 to 55 seconds, the novel RSS remarkably reduces the handover failures compared to the existing RSS. The robustness of the novel RSS is in the way of decision making process which is based on the BS' RSS accumulative values during *n*-time intervals, whereas, the existing RSS is dependent on one RSS measurement, which makes it more susceptible to signal fluctuating, and at the same time, it provides a high accuracy ratio when compared to the existing RSS. The novel RSS's missed handovers are considered few, despite being 7, when compared to the existing RSS approaches with more than 20 missed handovers.

As a conclusion from Figures 4 and 5, the enhanced GPS approach has higher accuracy compared to the novel RSS prediction approach. On the contrary, the RSS approach shows less power consumption, no additional data requirements, and lower cost. The reason behind proposing these two prediction approaches is to add flexibility to the prediction process based on the user's preferences. The user tradeoffs are among accuracy, power consumption, and cost of prediction.

### 3.2. Overall Scenario

**Figure 6:**
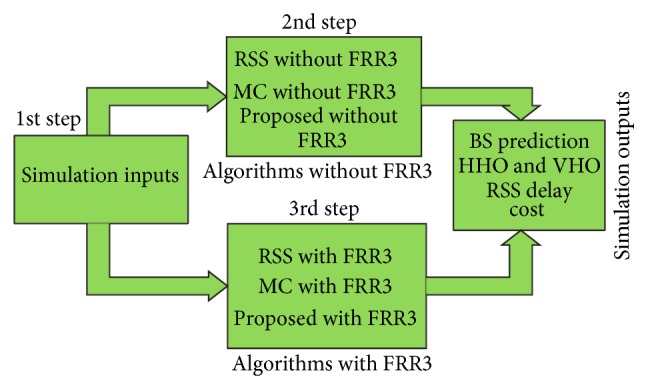
The simulation steps.

Figure 6 expresses the simulation steps series. The simulation sequence runs as the following steps.

- Input parameters are set according to Table 1.
- The existing RSS, multiple criteria (MC) approach, and proposed algorithm (modified multiple criteria approach with two handover thresholds) are presented.
- FRR3 technique is implemented to all the approaches.

All the approaches proposed above have been simulated, tested, and evaluated for BS prediction, HHO and VHO, RSS, delay, and cost as simulation outputs. The details result and the decision of each approach are described below.

#### 3.2.1. Prediction of Target BS and Target Technology

**Figure 7:**
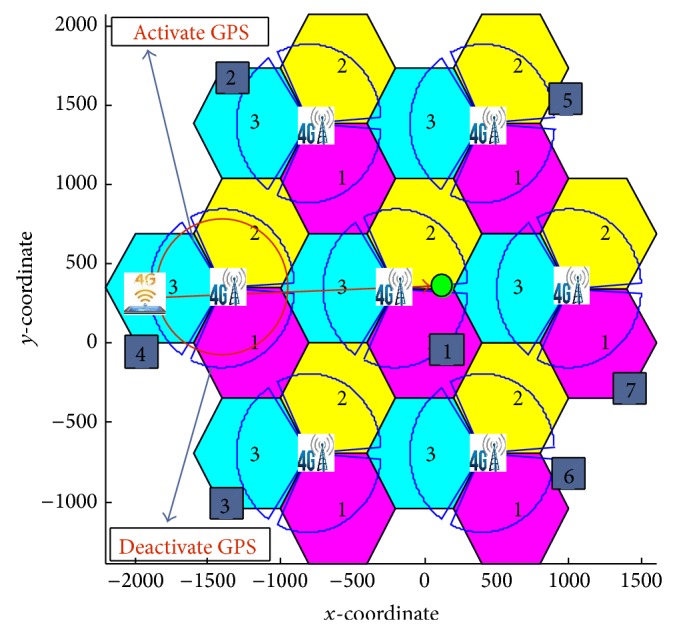
MS movement in the overall scenario simulation.

In this section, the mechanism's behavior for the prediction and selection of the most appropriate target BS and technology is investigated. The topology of Figure 7 consists of 7 BSs; each BS supports two technologies (LTE and WiMAX). The results being studied are comprised of three approaches, with and without the FRR3 technique.

The studied approaches are named as follows: existing RSS approach, MC approach, and the proposed approach. The weighting factors for each criterion (user's preferences) are applied as inputs to the proposed approach. From Figure 7, we assume that the MS is roaming from BS 4 to BS 1. Based on the user's preference (Table 1), the enhanced GPS prediction approach is chosen as the prediction approach since the cost weight is only 5% as opposed to the novel RSS approach.

**Figure 8:**
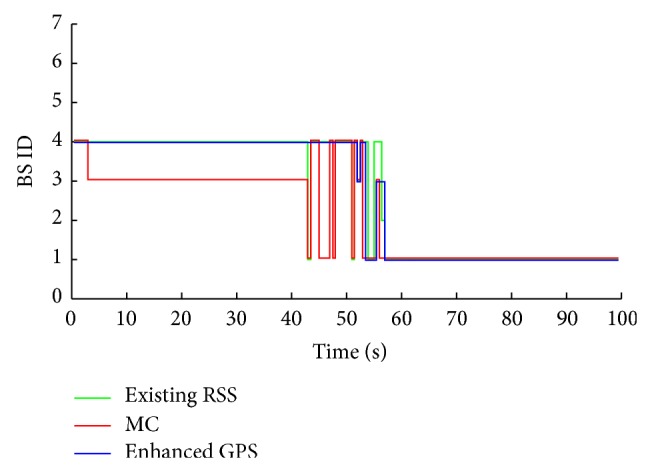
BS prediction without FRR3.

A comparison of the results of the enhanced GPS approach with other competitive approaches regarding target BS prediction without FRR3 is presented in Figure 8. During the simulation period, the enhanced GPS approach proves an accurate BS prediction and high stability compared to the other two approaches. It shows only two handover failures (connection to BS 3 instead of BS 1). Since the MS experiences high ICI and CCI at BS 1 (central BS) and BS 3 seems to be having more acceptable factors than BS 4, the MC approach connects to BS 3 instead of 4 for quite a long time (keep MS without connection between 3 and 43 seconds). Therefore, this kind of prediction is not convenient or practical for utilization in high attenuation areas. The existing RSS approach shows average redundant handovers ratio between the MC and the enhanced GPS approach.

**Figure 9:**
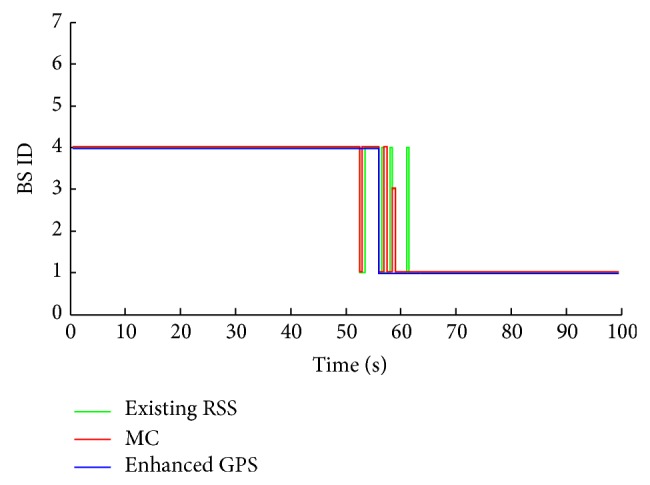
BS prediction with FRR3.

**Figure 10:**
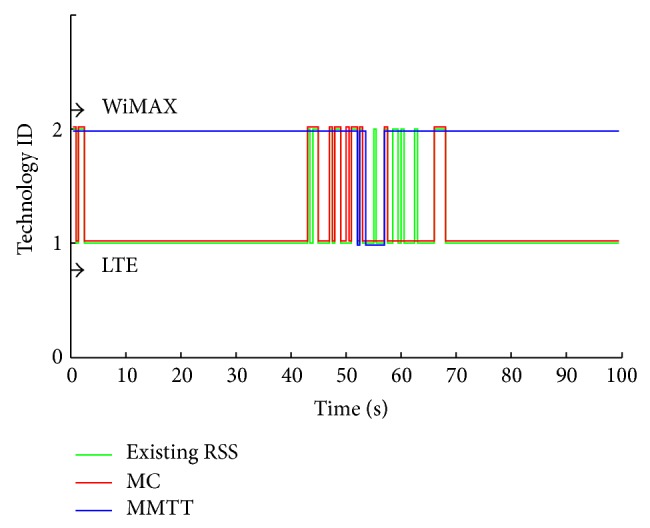
HHO and VHO without FRR3.

**Figure 11:**
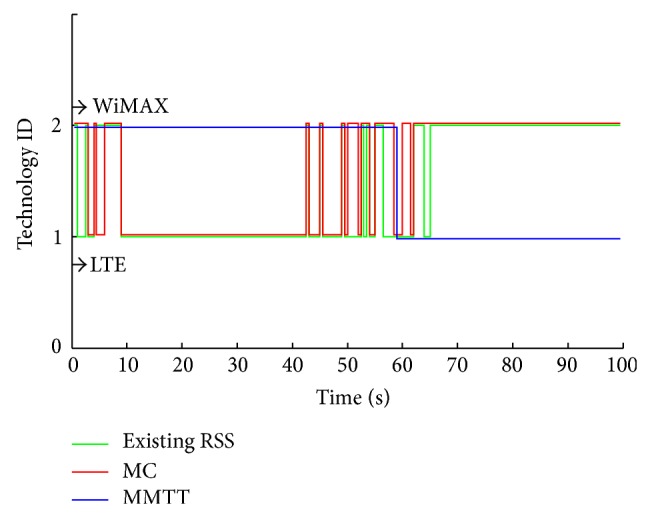
HHO and VHO with FRR3.

The three approaches show a significant enhancement in the prediction process by applying the FRR3, as can be seen clearly from Figure 9 where the enhanced GPS approach provides no redundant handovers. The MC's efficiency has increased remarkably since it is converted to a valid connection with few redundant handovers, that is, from 0–60 seconds. High handovers stability leads to more efficiency for exploiting the available resources from both technologies (LTE and WiMAX). That is shown in Figures 10 and 11, where the MMTT algorithm shows the highest value of the handover quality indicator compared to the other two approaches. The other approaches show many unwanted handovers and waste available resources since they reserve and use a lot of resources from both technologies for a too short period inefficiently. As it can be observed, the degradation of MS' QoS is a conclusion for both of the existing RSS and MC.

**Figure 12:**
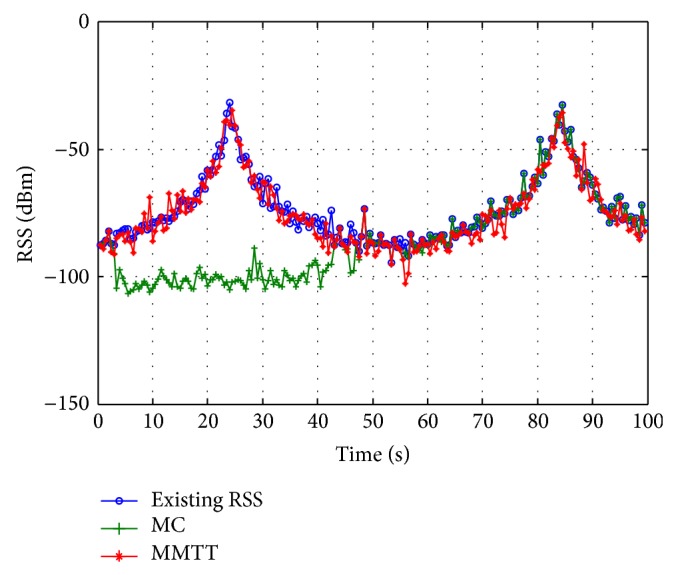
Received signal strength without using FRR3.

**Figure 13:**
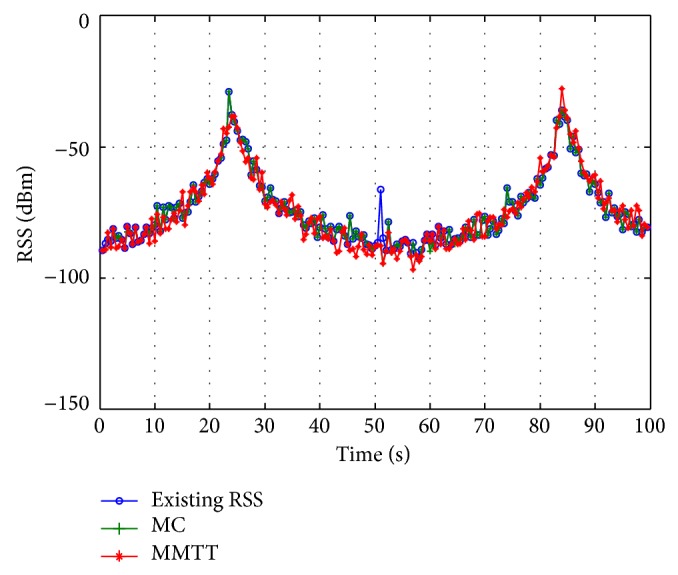
Received signal strength with FRR3.

The MMTT approach keeps the signal strength within an acceptable range during the whole simulation period except at the 57th second (Figure 12). As illustrated in Figures 12 and 13, it is obvious that there is remarkable enhancement between the received signal strength before and after applying the FRR3 technique. At the 57th second, the MMTT algorithm is recovered from degradation in the received signal strength to an acceptable level. At the same time, from 4 to 41 seconds, the MC approach demonstrates a huge enhancement, since it is converted from insufficient to sufficient handover approach.

**Figure 14:**
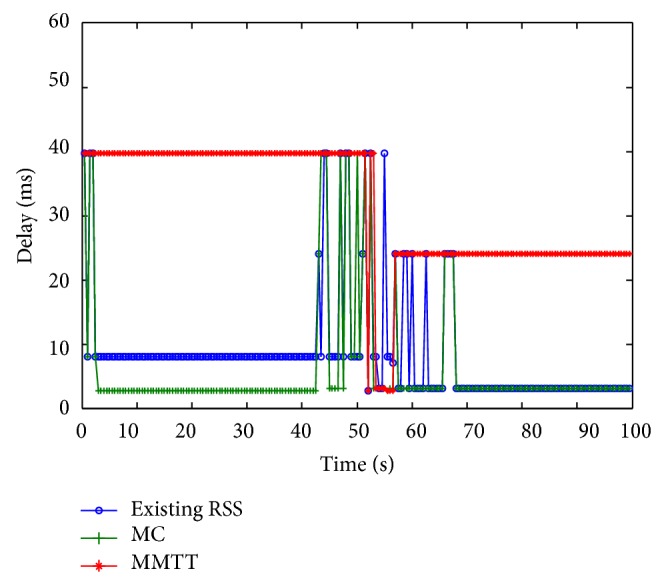
Delay without FRR3.

**Figure 15:**
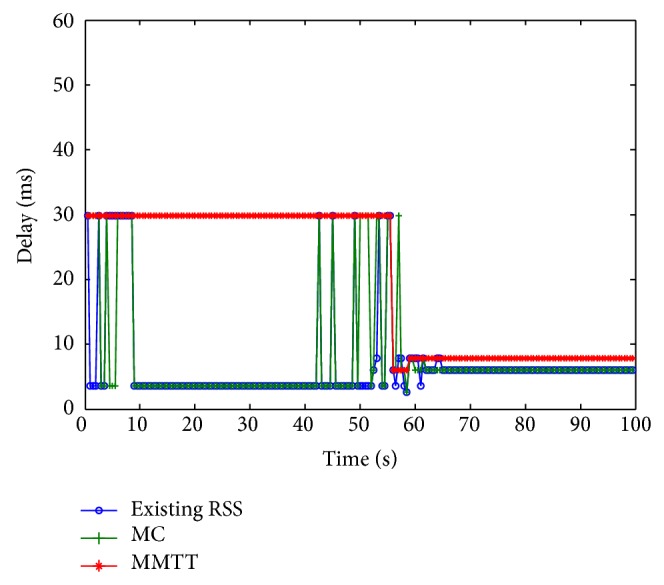
Delay with FRR3.

Even the MMTT approach has the highest handover delay compared to the other two approaches, but it still satisfies the user's preferences (Figure 14). The FRR3 decreases the handover process delay of the MMTT approach up to roughly 15% in comparison to the scenario with no FRR3 (Figure 15). This means that the proposed algorithm determines the target BS and technology faster than without using the FRR3 technique.

**Figure 16:**
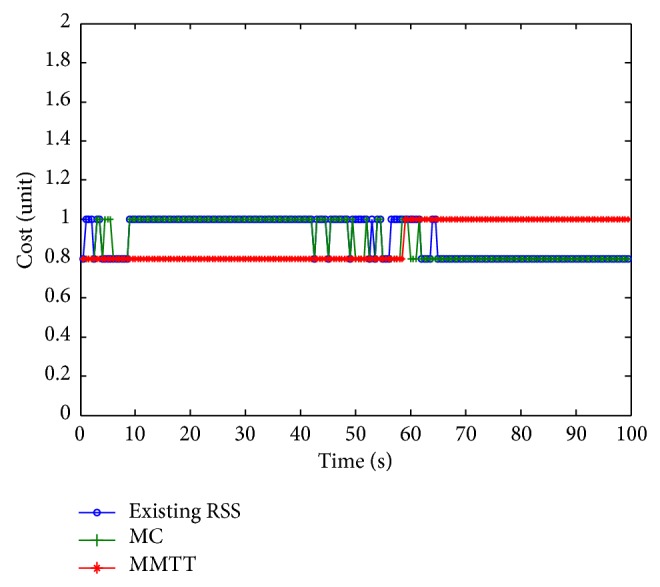
Connection cost.

From Figure 16, it is surmised that the proposed algorithm achieves the lowest average cost value equal to 0.883, while MC results in 0.8915, and the existing RSS has the highest value of 0.9.

## 4. Conclusions

In this paper, the enhancement of GPS and RSS algorithms by adding the virtual threshold (trigger threshold) is presented. In the RSS algorithm, a novel “*n*” number of measurements method is used instead of the standard one measurement technique. Therefore, the RSS approach becomes less affected by the interferences from the surrounding BSs, whereas in the GPS algorithm, the enhancement decreases the cost and power consumption due to the fact that the GPS device is only active between the trigger and handover thresholds instead of all the time.

Moreover, we demonstrate a modified multiple criteria with two thresholds algorithm resulting in an increase in the efficiency of target network selection. The selection is based on the user preferences since it uses the self-learning algorithm to determine the trigger and handover thresholds dynamically. Finally, by adding the FRR3 technique to the system, the efficiency of the prediction of target BS and the selection of target technology is increased, and the delay is decreased by approximately 15%. As far as we know, this is the first investigation of a setup combining target BS prediction approach, technology selection approach, and FRR3 technique.

A further enhancement may be added to this work, such as a generic and extensible media access control layer (MAC) for the networks. This enhancement will allow the MS to have a smoother transmission and an ability to receive data among different BSs with different network types. Since the MS will be supported with smooth connectivity with different BSs technologies, and, not requiring any extra equipment, the system is expected to have less delay, lower cost, and better performance.

## Figures and Tables

**Algorithm 1:**
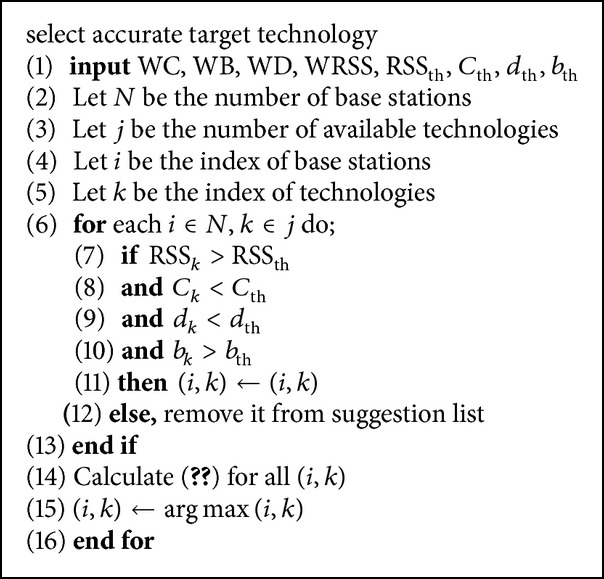
Evaluation of the BS' technology.

**Table 1:** Overall scenario of simulation parameters.

Input parameters	Units	Values
Number of cell		7
Number MS		1
RSS weight		50%
Bandwidth weight Cost weight Delay weight		20% 5% 25%
Angle of movement	Degree	0
Mobility type		PRWMM probabilistic random waypoint mobility model
Path loss type	dB	Macro urban path loss PL = 33.81 × log⁡10(fc) − 79.4 + 3 + 35.04 × log⁡10(d)
Simulation time	Second	100
MS highest	Meter	1
BS highest	Meter	40
